# Evaluation of the Esthetic Properties of Developmental Defects of Enamel: A Spectrophotometric Clinical Study

**DOI:** 10.1155/2015/878235

**Published:** 2015-03-22

**Authors:** Fabrizio Guerra, Marta Mazur, Denise Corridore, Debora Pasqualotto, Gianna Maria Nardi, Livia Ottolenghi

**Affiliations:** Department of Oral and Maxillo-Facial Sciences, “Sapienza” University of Rome, Via Caserta 6, 00161 Rome, Italy

## Abstract

*Objectives*. Detailed clinical quantification of optical properties of developmental defect of enamel is possible with spectrophotometric evaluation. Developmental defects of enamel (DDE) are daily encountered in clinical practice. DDE are an alteration in quality and quantity of the enamel, caused by disruption and/or damage to the enamel organ during amelogenesis. *Methods*. Several clinical indices have been developed to categorize enamel defects based on their nature, appearance, microscopic features, or cause. A sample of 39 permanent teeth presenting DDE on labial surface was examined using the DDE Modified Index and SpectroShade evaluation. The spectrophotometric approach quantifies *L* ^*^ (luminosity), *a* ^*^ (quantity of green-red), and *b* ^*^ (quantity of blue-yellow) of different DDE. *Conclusions*. SpectroShade evaluation of the optical properties of the enamel defect enhances clinical understanding of severity and extent of the defect and characterizes the enamel alteration in terms of color discrepancy and surface characterization.

## 1. Introduction

Developmental defects of enamel (DDE) are daily encountered in clinical practice. DDE are alteration in quality and quantity of the enamel, caused by disruption and/or damage to the enamel organ during the amelogenesis process. The clinical aspect of the defect depends on the stage of development during which the insult occurs as well as the extent and duration of the insult. Enamel hypoplasia (HE) is a quantitative defect and presents a scarce enamel thickness, while enamel hypomineralization (EO) is a qualitative enamel deficiency presenting alterations in enamel translucency and opacity which may be diffuse (DIO) or demarcated (DEO) with white, yellow, or brown colour [1, 2]. DDE can have a significant impact on oral health and esthetics, tooth sensitivity, and altered occlusal functions [3, 4]. Enamel defects are also risk conditions for dental caries and erosion in children [5, 6]. Epidemiologic data (DDE prevalence in permanent dentition ranges from 10% to 49%) reflect an increasing trend of this condition, which should be considered as a public health problem and a challenge for dental practitioners. Several clinical indices have been developed to categorize enamel defects and they can be divided into (a) specific fluorosis indices (Dean/WHO, Thylstrup and Fejerskov, and TSIF indices), which identify and categorize only dental fluorosis, and (b) descriptive indices (the Al-Alousi and the Developmental Defects of Enamel Index, the Modified DDE Index), with no etiological assumption [2].

**Figure 1:**
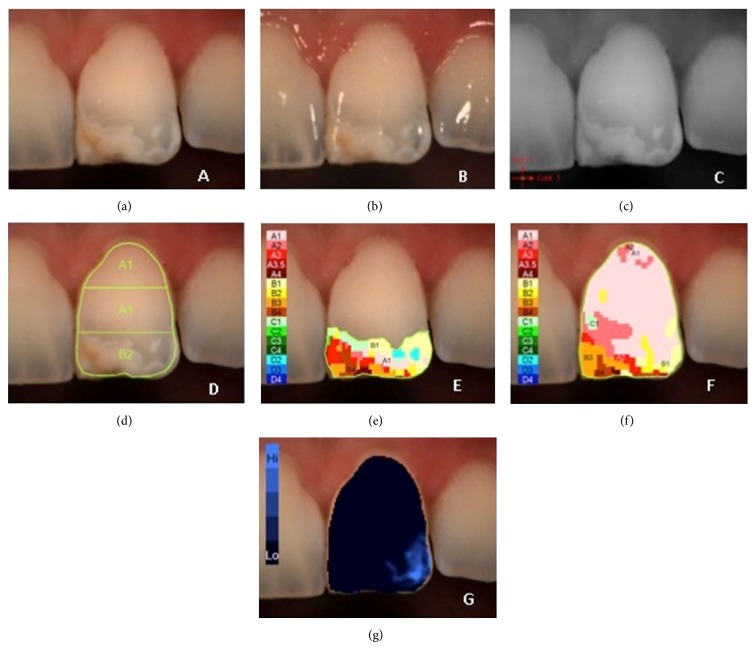
Example of *L* ^*^ *a* ^*^ *b* ^*^ measurements of demarcated opacity: (a) polarized image; (b) gloss mode allowing the identification of “pure enamel zones”; (c) contrast image; (d) evaluation of the three equal zones along the median axis: gingival, central, and incisal; (e) colour distribution and detailed mapping of the defect surface; (f) overall detailed mapping; (g) translucency.

**Figure 2:**
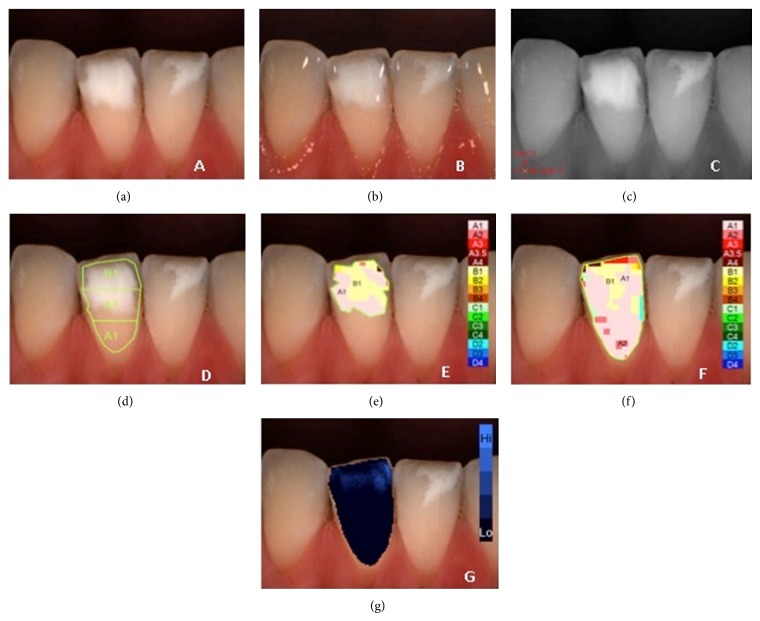
Example of *L* ^*^ *a* ^*^ *b* ^*^ measurements of demarcated opacity on lower central right incisor: (a) polarized image; (b) gloss mode allowing the identification of “pure enamel zones”; (c) contrast image; (d) evaluation of the three equal zones along the median axis: gingival, central, and incisal; (e) colour distribution and detailed mapping of the defect surface; (f) overall detailed mapping; (g) translucency.

**Figure 3:**
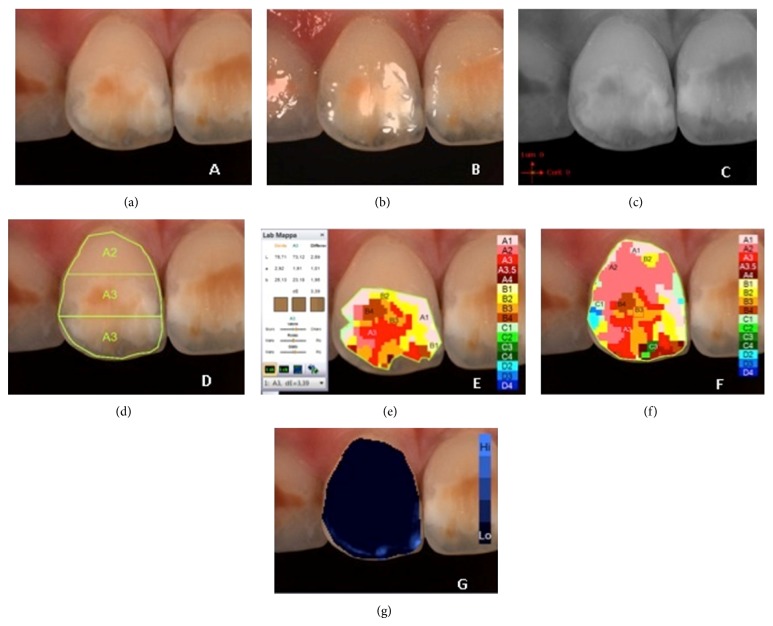
Example of *L* ^*^ *a* ^*^ *b* ^*^ measurements of demarcated opacity on upper central right incisor: (a) polarized image; (b) gloss mode allowing the identification of “pure enamel zones”; (c) contrast image; (d) evaluation of the three equal zones along the median axis: gingival, central, and incisal; (e) colour distribution and detailed mapping of the defect surface; (f) overall detailed mapping; (g) translucency.

**Figure 4:**
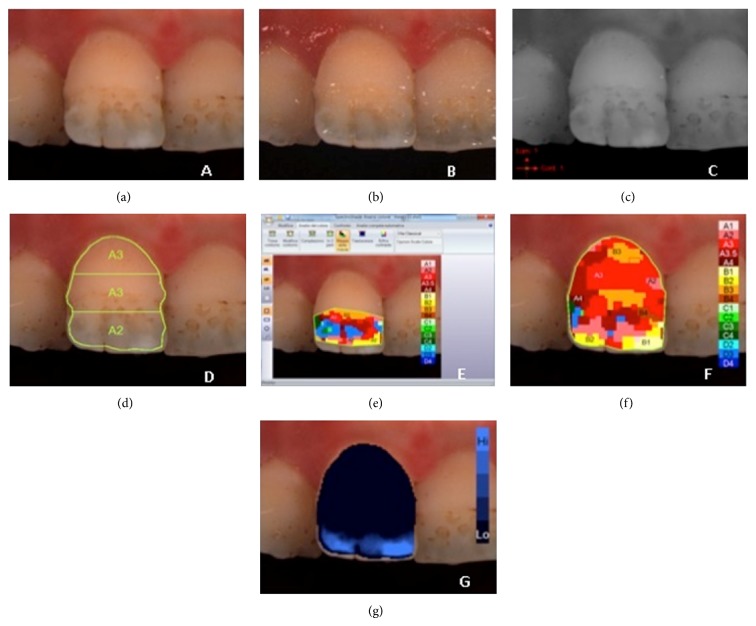
Example of *L* ^*^ *a* ^*^ *b* ^*^ measurements of hypoplastic defects on upper central incisor: (a) polarized image; (b) gloss mode allowing the identification of “pure enamel zones”; (c) contrast image; (d) evaluation of the three equal zones along the median axis: gingival, central, and incisal; (e) colour distribution and detailed mapping of the defect surface; (f) overall detailed mapping; (g) translucency.

**Figure 5:**
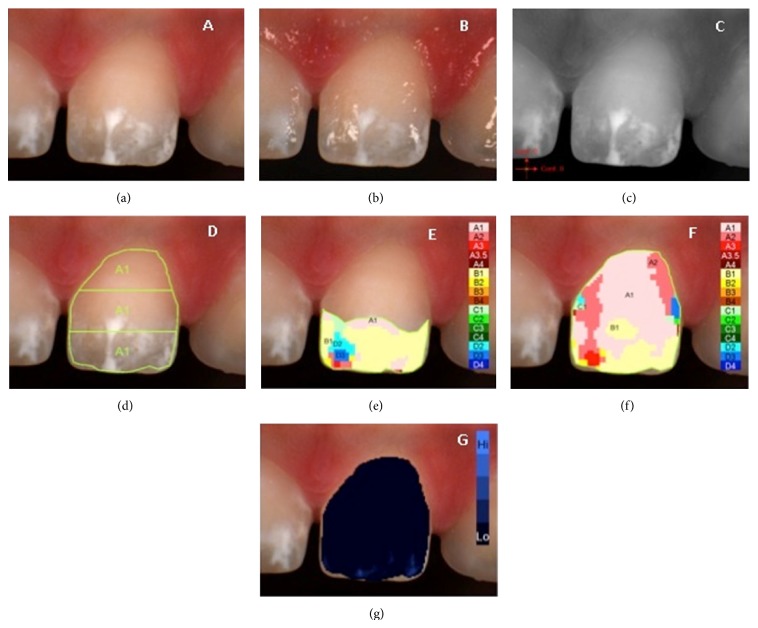
Example of *L* ^*^ *a* ^*^ *b* ^*^ measurements of diffused opacity on upper central incisor: (a) polarized image; (b) gloss mode allowing the identification of “pure enamel zones”; (c) contrast image; (d) evaluation of the three equal zones along the median axis: gingival, central, and incisal; (e) colour distribution and detailed mapping of the defect surface; (f) overall detailed mapping; (g) translucency.

The Modified DDE Index [7] is a descriptive index derived from the Developmental Defects of Enamel Index [8]. It is more practical and comparable index for epidemiological studies and it allows efficient recording of prevalence and severity of enamel defects. The criteria for classification are related with histopathological changes [9]. The DDE Modified Index divides defects into three types: demarcated (Figures 1, 2, and 3), diffuse (Figure 5), and hypoplastic (Figure 4). The diffuse opacity category probably contains most of the fluoride-related opacities. However, this group contains some nonfluoride opacities as well, and no attempt is made to differentiate between these types. The extent of the defect should be recorded in thirds of the tooth surface area and a limit in size of greater than 1 mm in diameter should be used to distinguish between normal and abnormal enamel defects (Table 1).

The aetiology of DDE is not completely clear. Genetic and hereditary factors such as in amelogenesis imperfecta are involved, along with acquired, systemic, and environmental factors such as fluoride intake and medications, nutritional deficiencies, prenatal infections, or chicken pox or other early childhood diseases [3, 10–12].

Clinical detecting of DDE occurs in children, adolescents, and young adults. DDE on vestibular surface of upper and lower arch may cause the patient distress, while parents are concerned about esthetic problem and ask for solution. Clinical evaluation of defect severity and extent is part of the esthetic management of these lesions and may influence the treatment option choice. The aim of this paper is to develop a clinical approach based on spectrophotometric measurements. Subjective human perception of colour is susceptible to bias. It is possible to exclude this bias by using spectrophotometer that allows an objective, quantitative colorimetric method and can be used under routine clinical conditions [13, 14]. Spectrophotometric measurement generates quantitative data not only of the defects area, but also of the surrounding sound enamel surface. These data can be used for the quantification of esthetic properties of the DDE defects classified by DDE Modified Index.

## 2. Material and Methods

The study design was not reviewed by the Dental School's Ethics Committee, because instrumental and observational analyses are usual clinical educational activities at the Sapienza University. Prior to each measurement, teeth were cleaned with a prophylaxis paste and rinsed with water spray. Teeth dehydration was controlled to avoid shade changes due to humidity loss. The study population was of 30 subjects, in the age range of 13–19 years (mean age: 15,3); 39 teeth were analyzed. Of the enrolled patients 51,5% were girls and 48,5% were boys. There were no gender related differences between the three DDE modified index groups distributions (demarcated, diffuse, and hypoplastic).

A digital camera (Nikon D90) with a macro lens (105 mm Macro lens, Nikon) and a macro flash (R1C1 Macro flash, Nikon) documented the developmental defects of enamel on the labial surface of central and lateral, upper and lower incisors and upper and lower canines. The digital photo was used to classify the enamel defect consistently with DDE Modified Index.

In this study a calibrated reflectance spectrophotometer (SpectroShade, MICRO, Serial N HDL1407, MHT, Arbizzano di Negrar, Verona, Italy) was used. The position of the device is perpendicular to the labial surface of the clinical crown and it is reproducible in order to obtain always equal measurement conditions. The D65 light source (6500°K) illuminates each tooth simultaneously from two sides at 45° angle. The system has two detector areas (both 18 mm × 13 mm) where the reflected light is directed at 0°. One detector (color CCD chip) generates the color video image; the other (b/w CCD detector) records the spectrophotometric data. The stored data are used to create detailed CIE *L* ^*^ *a* ^*^ *b* ^*^ data of the tooth surface.

MHT spectrophotometer analyzes the dental surface every 8 nm and allows a large number of different data representations on specific tooth area. All the clinical factors influencing esthetic appearance of the teeth are taken into consideration with this method [15]. The MHT software divides the vestibular tooth area into three equal zones along the median axis: the same method is described by DDE Modified Index during tooth area examination. These well-defined enamel areas have different optical properties in sound tooth and the spectrophotometric evaluations show how CIE *L* ^*^ *a* ^*^ *b* ^*^ coordinates vary along the median axis from higher gingival point to the incisal edge [16]. Enamel is more translucent and, in respect to tooth color, it plays only a minor role through scattering at wave lengths in the blue range, while dentin is more opaque and, according to ten Bosch and Coops [17], determinates mainly the color of the tooth. All SpectroShade assessments were performed by one trained operator.

Consistently with DDE Modified Index, a more meaningful way to present data was to group the defects into three broad categories, that is, demarcated opacities (DEO), diffuse opacities (DIO), and hypoplastic defects (HE), with provision to record discolouration and any other defects. The demarcated opacities can present white/cream and yellow/brown shades. The diffuse opacities can be like fine delicate lines, or patchy, or confluent opacities. The diffuse opacities tend to fade into the surrounding enamel. These defects can be present on tooth surface singularly or in combination.

To define the defect in relation to the extent of the tooth surface covered by the defect, the vertical length of the measured teeth was divided into three equal zones along the median axis. In each zone all the area was detected and defined by using the device software. The *L* ^*^ value (*y*-axis) is a measure of the lightness of an object on a scale ranging from 0 (black) to 100 (white). The *a* ^*^ value is a measure of redness (positive *a* ^*^) or greenness (negative *a* ^*^). The *b* ^*^ value is a measure of yellowness (positive *b* ^*^) or blueness (negative *b* ^*^). The *a* ^*^ and *b* ^*^ coordinates approach zero for neutral colours (white, greys) and increase in magnitude for more saturated or intense colour [18–21].

## 3. Results

### 3.1. Descriptive Statistics

The defect distribution was 61,5% DEO (demarcated opacities), 20,5% DIO (diffuse opacities), and 18% HE (enamel hypoplasia). The more frequent localization of the developmental defects of enamel on the tooth surface was on the incisal area (58,9%), then on the incisal and central areas (33,4%), on all the clinical crown surface (5,1%), and in few cases (2,6%) on the central tooth area. No one defect was present exclusively on the gingival area.

The mean value of *L* ^*^ of sound enamel was 72,44; the mean value of *a* ^*^ was 6,46; the mean value of *b* ^*^ was 19,47, respectively.

The results of the spectrophotometric measurements show the following.

*(i) In the DEO (Demarcated Opacities).* The mean value of *L* ^*^ of the defect was 69,71. The mean value of *a* ^*^ was 2,80. The mean value of *b* ^*^ was 15,58.

*(ii) In the DIO (Diffuse Opacities).* The mean value of *L* ^*^ of the defect was 69,87. The mean value of *a* ^*^ was 3,33. The mean value of *b* ^*^ was 11,46.

*(iii) In the HE (Hypoplasia).* The mean value of *L* ^*^ of the defect was 65,28. The mean value of *a* ^*^ was 10,65. The mean value of *b* ^*^ was 21,42.

The descriptive statistics for mean *L* ^*^ *a* ^*^ *b* ^*^ values is illustrated in Table 2.

Table 3 shows the enamel defect type (DEO, DIO, and HE), localization along the median axis (incisal, central, or gingival), and the Vita 3D Master shade selection measured by MHT spectrophotometer software, respectively, of sound and defect enamel surface.

## 4. Discussion

Little is known about the optical properties of developmental defects of enamel in a young population. Clinical detecting of DDE is more frequent everyday but there is a lack of information about the optical properties of the defect surface and the surrounding sound enamel surface.

The tooth optical properties describe a complex phenomenon, which can be even more complex if a developmental defect of enamel is present on labial tooth surface. Value, hue, and chroma describe tooth colour, but there are more subtle secondary optical properties that affect the overall tooth appearance: translucency, opalescence, opacity, iridescence, surface gloss, and fluorescence [20, 21]. Translucency, opacity, and opalescence have been viewed as the most important indicators of the quality and quantity of light reflection [22].

In this study the tooth labial surface was divided into three equal zones (incisal, central, and gingival) along the median axis and the aspects we consider most important for tooth perception, that is, *L* ^*^, *a* ^*^, and *b* ^*^ (resp., the amount of luminosity, green/red, and blue/yellow), were analyzed. Sound enamel surface and affected surface were analyzed using the same spectrophotometric method.

The developmental defect of enamel can be localized at the cervical, middle, or incisal area of the tooth. In a sound tooth these areas have different optical properties, because of the structure complexity and variability of enamel thickness and the below dentine. Some considerations can be illustrated about the optical properties of a sound tooth. Hasewaga has measured *L* ^*^ *a* ^*^ *b* ^*^ in 5 locations along the median axis and found significant variation. *L* ^*^ is highest in the center zone (*L* ^*^ = 73) and becomes lower in the gingival zone (*L* ^*^ = 69) and more lower towards the incisal edge (*L* ^*^ = 64); the highest *a* ^*^ value is in the gingival area (*a* ^*^ = 8,5) and it gradually becomes lower towards the incisal edge (*a* ^*^ = 2,0); *b* ^*^ is highest in gingival region (*b* ^*^ = 20) and gradually decreases towards the incisal edge (*b* ^*^ = 13) [23]. The existing differences between these zones are statistically and clinically significant also for detecting and describing enamel colour alteration when developmental defects are present. Translucency indicates the quality and quantity of light reflection and it decreases from incisal towards central tooth area.

In our opinion, the less the defect is integrated within the surrounding sound enamel surface, the more the DDE is clinically evident, this condition increasing from gingival to incisal tooth area. Loss of surface gloss affects the tooth vitality appearance. When a demarcated opacity is present on the incisal zone with white, yellow, and/or brown characterization, the surface gloss is completely altered. Developmental defects of enamel interest also tertiary anatomy. The tertiary anatomy is defined by vertical, horizontal, and varied textures. Marginal ridges and developmental lobes define the vertical texture, while fine, transverse, and delicate wavelike grooves define the horizontal characterization. The horizontal grooves are called perikymata. Tertiary anatomy of a sound maxillary central incisor is well pronounced in young population, while with age it is lost due to horizontal and vertical wear. The tertiary anatomy is completely erased when demarcated opacities are present on the tooth surface. Enamel hypoplasia such as pitted enamel hypoplasia often can affect also vertical tertiary anatomy, determining in the meanwhile a complete aberration of tertiary characterizations.

Spectrophotometric analysis presents a lot of advantages: it excludes bias due to subjective evaluation and analyses every 1–10 nm of the visible spectrum and the collected data are accurate. Incisal, central, and gingival zones have really different CIE *L* ^*^, *a* ^*^, and *b* ^*^, because of changing thickness in enamel and dentine layers. Moving from the incisal zone to the gingival, the tooth thickness increases and opacity and *a* ^*^ values increase too, while luminosity (*L* ^*^ values) decreases. The gingival zone has been shown to have the lowest translucency [23] and significantly higher *a* ^*^ values; *b* ^*^ values slightly increase with thickness, in a constant and linear way [19]. According to Modified DDE Index, a defect can be considered greater if the covered surface is more than 1 mm of diameter. In our opinion the defect localization on the three previously described zones (incisal, central, and gingival) is an important factor for the defect characterization. Optical properties of the defect have to be confronted with the optical properties of the corresponding sound enamel zone and the more they diverge in *a* ^*^ and *b* ^*^ values, the more is the perception of the existing contrast. For example, as gingival zone has the lowest translucency, higher *a* ^*^ values, and increased *b* ^*^ values but decreased luminosity (*L* ^*^ values), a defect with similar optical properties will be more accepted in this zone, while the same defect in the incisal zone will appear more evident due to higher differences in optical properties with the interested zone.

A recent study presented a correlation between the colour of enamel and the severity of hypomineralization, where yellow and brown colour of the hypomineralized enamel was at a higher risk for PEB (posteruptive breakdown) compared with white defects [24].

## 5. Conclusions

The analysis of the interaction of light with dental structures is really important. A novel quantitative in vivo approach for characterization of developmental defects of enamel optical parameters according to DDE Modified Index was developed during this study and it proved its feasibility on a limited number of patients. The spectrophotometric evaluation in this study required 5 minutes for each patient. The application of this method on a larger number of subjects may allow for a clinical correlation between colorimetric features and clinical severity and between diagnosis and therapeutic decision making process.

## Figures and Tables

**Table 1:** 

Basic type of DDE	Subtype of DDE
Demarcated opacities (DEO)	Demarcated opacities (white/cream)
	Demarcated opacities (yellow/brown)

Diffuse opacities* * (DIO)	Diffuse opacities lines/patchy
	Diffuse opacities confluent
	Confluent/patchy stain gloss of enamel

Hypoplasia (HE)	Hypoplasia pits
	Hypoplasia missing enamel

Discolouration	

**Table 2:** 

Sample	Type	*L*	*a*	*b*
1	DEO	69,35	2,26	19,84
2	DEO	68,22	2,89	21,26
3	DEO	71,40	4,40	24,68
4	DEO	70,91	1,47	12,17
5	DEO	65,42	3,95	16,39
6	DEO	62,50	9,34	24,35
7	DEO	79,82	1,21	9,30
8	DEO	74,94	1,51	9,29
9	DEO	68,65	7,70	28,42
10	DEO	67,36	3,03	16,04
11	DEO	65,67	3,21	17,38
12	DEO	74,13	1,54	12,95
13	DEO	66,99	1,45	10,58
14	DEO	69,77	0,82	10,78
15	DEO	64,77	8,43	16,33
16	DEO	63,55	8,61	24,74
17	DEO	75,90	1,02	14,27
18	DEO	67,28	1,18	11,46
19	DEO	65,12	0,54	12,15
20	DEO	72,30	0,85	21,05
21	DEO	74,18	1,36	7,37
22	DEO	69,26	1,17	15,26
23	DEO	69,21	0,12	8,81
24	DEO	76,29	−0,73	9,13

	Mean	69,71	2,80	15,58

25	DIO	73,18	3,24	10,34
26	DIO	72,17	0,91	8,41
27	DIO	67,57	2,12	10,81
28	DIO	66,80	2,74	11,68
29	DIO	69,39	3,13	9,21
30	DIO	66,59	6,60	14,97
31	DIO	68,75	4,58	16,10
32	DIO	74,49	3,35	10,18

	Mean	69,87	3,33	11,46

33	HE	70,34	10,74	18,98
34	HE	70,14	10,02	18,64
35	HE	62,64	5,20	21,84
36	HE	63,44	3,79	18,61
37	HE	65,01	9,84	18,34
38	HE	58,67	25,56	37,50
39	HE	66,69	9,40	16,01
	Mean	65,28	10,65	21,42

**Table 3:** 

Sample	Enamel defect		Shade (Vita 3D Master)	
	Type	Localization	Defect	Sound enamel
1	DEO	Incisal	1M1	3M1
2	DEO	Incisal/central	2R2,5	1M1
3	DEO	Incisal/central	3M3	2M1
4	DEO	Incisal	1M1	1M2
5	DEO	Incisal	5M1	3M2
6	DEO	Incisal/central	5M3	3M1
7	DEO	Incisal/central	0M1	1M2
8	DEO	Incisal	0M3	1M2
9	DEO	Incisal/central	3M3	3M3
10	DEO	Incisal	1M1	3R1,5
11	DEO	Incisal	2R1,5	3L1,5
12	DEO	Incisal	2M1	4M1
13	DEO	Incisal	1M1	2M1
14	DEO	Incisal	1M1	3M1
15	DEO	Incisal/central/gingival	3R1,5	4M1
16	DEO	Incisal/central	5M2	4M1
17	DEO	Incisal	2M1	3M1
18	DEO	Incisal	1M1	3M1
19	DEO	Incisal	2M1	3M1
20	DEO	Incisal	2R2,5	2M1
21	DEO	Incisal	1M1	2M1
22	DEO	Incisal	1M2	2M2
23	DEO	Incisal	2M1	2M1
24	DEO	Incisal	0M1	3M1
25	DIO	Incisal/central	1M1	2M1
26	DIO	Incisal	5M1	2R1,5
27	DIO	Incisal	0M1	3M1
28	DIO	Incisal	2M1	4M1
29	DIO	Incisal	2M1	4M1
30	DIO	Incisal/gingival	3M1	3M1
31	DIO	Incisal/central	3M1	3R1,5
32	DIO	Incisal/central	1M1	2M1
33	HE	Incisal/central	3M2	3M1
34	HE	Incisal/central	5M1	3M1
35	HE	Incisal	5M2	4M1
36	HE	Incisal	4M1	4R2,5
37	HE	Incisal/central	5M1	4M1
38	HE	Central	5M3	3M2
39	HE	Incisal/central	3R1,5	3M1
